# Micro- and Nanosecond Pulses Used in Doxorubicin Electrochemotherapy in Human Breast and Colon Cancer Cells with Drug Resistance

**DOI:** 10.3390/molecules27072052

**Published:** 2022-03-22

**Authors:** Nina Rembiałkowska, Vitalij Novickij, Dagmara Baczyńska, Magda Dubińska-Magiera, Jolanta Saczko, Julia Rudno-Rudzińska, Magdalena Maciejewska, Julita Kulbacka

**Affiliations:** 1 Department of Molecular and Cellular Biology, Faculty of Pharmacy, Wroclaw Medical University, Borowska 211A, 50-556 Wroclaw, Poland; nina.rembialkowska@umw.edu.pl (N.R.); dagmara.baczynska@umw.edu.pl (D.B.); jolanta.saczko@umw.edu.pl (J.S.); 2 Institute of High Magnetic Fields, Vilnius Gediminas Technical University, LT-03227 Vilnius, Lithuania; vitalij.novickij@vgtu.lt; 3 Department of Animal Developmental Biology, Faculty of Biological Science, University of Wroclaw, Sienkiewicza 21, 50-335 Wroclaw, Poland; magda.dubinska-magiera@uwr.edu.pl; 4 2nd Department of General Surgery and Surgical Oncology, Wroclaw Medical University, Borowska 213, 50-556 Wroclaw, Poland; julia.rudno-rudzinska@umw.edu.pl; 5 Laboratory of Experimental Anticancer Therapy, Hirszfeld Institute of Immunology and Experimental Therapy, Rudolfa Weigla 12, 53-114 Wroclaw, Poland; magdalena.maciejewska@hirszfeld.pl

**Keywords:** drug resistance, human adenocarcinoma, doxorubicin, electroporation, drug delivery

## Abstract

(1) Background: Pulsed electric field (PEF) techniques are commonly used to support the delivery of various molecules. A PEF seems a promising method for low permeability drugs or when cells demonstrate therapy resistance and the cell membrane becomes an impermeable barrier. (2) Methods: In this study, we have used doxorubicin-resistant and sensitive models of human breast cancer (MCF-7/DX, MCF-7/WT) and colon cancer cells (LoVo, LoVoDX). The study aimed to investigate the susceptibility of the cells to doxorubicin (DOX) and electric fields in the 20–900 ns pulse duration range. The viability assay was utilized to evaluate the PEF protocols’ efficacy. Cell confluency and reduced glutathione were measured after PEF protocols. (3) Results: The obtained results showed that PEFs significantly supported doxorubicin delivery and cytotoxicity after 48 and 72 h. The 60 kV/cm ultrashort pulses × 20 ns × 400 had the most significant cytotoxic anticancer effect. The increase in DOX concentration provokes a decrease in cell viability, affected cell confluency, and reduced GSSH when combined with the ESOPE (European Standard Operating Procedures of Electrochemotherapy) protocol. Additionally, reactive oxygen species after PEF and PEF-DOX were detected. (4) Conclusions: Ultrashort electric pulses with low DOX content or ESOPE with higher DOX content seem the most promising in colon and breast cancer treatment.

## 1. Introduction

Doxorubicin (DOX) or adriamycin is one of the most known and used cytostatic drugs. It is a chemotherapeutic form of the group of anthracyclines, which derive from *Streptomyces peucetius* [1]. The first data concerning the origin of DOX were noted in 1960, and there was observed anticancer activity against leukemia. The main action of doxorubicin is the inhibition of cell growth by topoisomerase 2. Up to now, DOX is still widely used in clinical practice for the treatment of numerous cancers, i.e., breast cancer, ovarian, prostate, stomach, liver, acute lymphocytic leukemia (ALL), acute myeloid leukemia (AML), or small lung cancer. Despite the broad anticancer activity, DOX reveals strong cardiotoxic effects [2,3]. Thus, there is still a need to improve doxorubicin activity with diminished side effects. Recently, novel methods for DOX delivery [4] have been implemented as nanocarriers [5], liposomes [6], or combinations of DOX with other drugs [7]. Among these methods, electroporation (EP) seems a good solution to improve drug transport [8,9]. The application of EP requires optimization for the particular types of cancer. The most commonly used is the ESOPE protocol [10], which is well established and successfully applied in clinics as electrochemotherapy (ECT) [11,12]. Here, we propose shorter pulses and higher voltages to enhance the doxorubicin effect in drug-resistant cells. The therapeutic efficacy of chemotherapy in many types of tumors is dramatically hindered by multidrug resistance (MDR). Our previous study, where ESOPE-based protocols with DOX or other drugs were used, indicated a promising usage in colon cancer [13,14], gastric [15], and breast cancer cells [16].

The improvement and optimization of other EP parameters are crucial, particularly in the resistant type of cancers. The other studies showed that ultrashort electric pulses could overcome drug resistance phenomena [15,17,18,19,20,21]. Here, two types of cancer cells were used: colon and breast cancer, including resistant counterparts. This study aimed to validate nanosecond and microsecond pulses for DOX delivery and its optimization. The effects were monitored by cell viability, confluency, and oxidative stress measurements.

## 2. Materials and Methods

### 2.1. Pulsed Power Setup and PEF Protocols

The experimental setup consists of 3 kV, 100 ns–1 ms square-wave high-voltage pulse generator (VGTU, Vilnius, Lithuania), and a commercially available electroporation cuvette with a 1 mm gap between electrodes (Biorad, Hercules, CA, USA) was used. For a 20 ns pulse delivery, the PPG-20 generator (FID Technology, Burbach, Germany) was applied. The voltage that was applied to the cuvette was varied in the 0.14–6 kV range, corresponding to a 1.4–60 kV/cm electric field. The pulses were delivered in bursts of 200 at 1 kHz for the 100–400 ns, 5–10 kV/cm protocols and bursts of 200–1200 at 0.2 kHz frequency for 40/60 kV/cm × 20 ns protocols. For the final experiments, the following protocols were used: PEF1—10 kV/cm × 300 ns × 200; PEF2—40 kV/cm × 20 ns × 400; PEF3—60 kV/cm × 20 ns × 400. The 1.2 kV/cm × 100 μs × 8-microsecond pulses were used as a reference (PEF4). PEF4 protocol corresponds to the ESOPE standard applied in clinical practice [10,22].

### 2.2. Cell Culture

Four human cell lines were used: two breast and two colon cancer lines. The studies were performed in vitro on doxorubicin-sensitive (MCF-7/WT) cell line obtained from the Department of Tumor Biology, Comprehensive Cancer Center, Maria Sklodowska-Curie Memorial Institute in Gliwice (Gliwice, Poland); doxorubicin-resistant type (MCF-7/DX) of human breast adenocarcinoma was purchased in ATCC^®^ and stimulated for DOX resistance; doxorubicin-sensitive (LoVo) and doxorubicin-resistant (LoVoDX) type of human colon adenocarcinoma obtained from Ludwik Hirszfeld Institute of Immunology and Experimental Therapy, Polish Academy of Sciences (Wroclaw, Poland). MCF-7/WT and MCF-7/DX cells were grown in DMEM (Sigma, Poznan, Poland), supplemented with 10% fetal bovine serum (Lonza BioWhittaker, Bettlach, Switzerland) and penicillin/streptomycin (Sigma-Aldrich, Poznań, Poland). LoVoDX and MCF-7/DX were obtained from parental counterparts by the exposure to increasing concentrations of DOX according to the protocol [23]. LoVo and LoVoDX cells were maintained in Ham’s F-12 (Sigma, Poznan, Poland) supplemented with 10% fetal bovine serum (FBS, Lonza BioWhittaker, Bettlach, Switzerland) and 1% penicillin/streptomycin (Sigma, Poznan, Poland). Cell cultures were cultured as a monolayer on 25 and 75 cm^2^ flasks (Sarstedt, Germany), maintained in a humidified atmosphere at 37 °C and 5% CO_2_, and detached for the experiment’s trypsinization (trypsin 0.025% and EDTA 0.02% solution, Sigma, Poznan, Poland). Cells were passed every 2–3 days and a day before the experiment.

### 2.3. MDR Proteins Level by Western Blot Assay

Total protein fraction was extracted from cells using a lysis buffer (50 mM Tris-HCl, pH 8.0, 150 mM NaCl, 1% NP-40, 0.1% SDS, 0.5% sodium deoxycholate) supplemented with protease inhibitor cocktail and phenylmethylsulfonyl fluoride (Sigma-Aldrich, St. Louis, MO, USA). Protein concentration was determined by BCA method. Then, 50 µg heat-denatured protein samples were separated on 10% gels using SDS-PAGE and transferred onto a nitrocellulose membrane (Sigma-Aldrich, Poznan, Poland). After blocking with 5% non-fat dried milk solution, the membranes were incubated at 4 °C overnight with 1000-times-diluted mouse monoclonal antibodies against ABCB1 (PGP, MDR1) (Santa Cruz, sc13131), ABCC1 (MRP1) (Santa Cruz, sc-18835), and ABCG2 (BCRP) (Santa Cruz, sc-377176). Blots were developed using a Mouse ExtrAvidin Peroxidase Staining Kit (Sigma-Aldrich, Poznan, Poland) and Clarity Western ECL Substrate (BioRad, Warsaw, Poland). Chemiluminescent signal was detected on G-Box Chemi XRQ chemiluminescence imaging and gel documentation system (Syngene, Cambridge, UK) 

### 2.4. MDR Gene Expression by Real-Time PCR

Cells were seeded on Petri dishes and then incubated with anticancer agents diluted in the culture medium for 24 h. After that, the cells were rinsed with PBS, removed by trypsinization, and centrifuged (280× *g*, 5 min). The dry cell pellet was stored at −20 °C for further experiments. The total RNA was isolated using a NucleoSpin® RNA II kit (Macherey-Nagel GmbH & Co., Düren, Germany) following the manufacturer’s protocol. Reverse transcription reaction (RT) was performed using 600 ng of extracted total RNA and a High-Capacity cDNA Reverse Transcription Kit (Thermo Fisher Scientific, Waltham, MA, USA) in a final volume of 20 μL according to the manufacturer’s instructions. AceQ qPCR Probe Master Mix (Vazyme Biotech, Nanjing, Jiangsu, China) and specific TaqMan assays as follows: ABCB1- Hs00184500_m1; ABCC1- Hs00219905_m1; ABCG2- Hs01053790_m1; LRP1- Hs00233856_m1, and Hs99999905_m1 for glyceraldehyde-3-phosphate dehydrogenase; GAPDH (Thermo Fisher Scientific, Waltham, MA, USA) were used to assess RNA expression according to the manufacturers’ instructions. We added 3 μL of three-times-diluted RT products to a single real-time polymerase chain reaction (RT-PCR). All the reactions were performed in triplicate in 96-well plates under the following thermal cycling conditions: 5 min at 95 °C followed by 40 cycles of 10 s at 95 °C and 30 s at 60 °C. The reactions were run in the Optical Real-Time PCR Thermocycler (Biometra GmbH, Göttingen, Germany), and the threshold cycle data (Ct) were collected using qPCRsoft (Biometra GmbH, Göttingen, Germany). For the relative quantification (RQ), the samples were normalized against the expression of GAPDH mRNA using the ΔΔCT method.

### 2.5. PEFs and Doxorubicin Exposure

**Figure 1:**
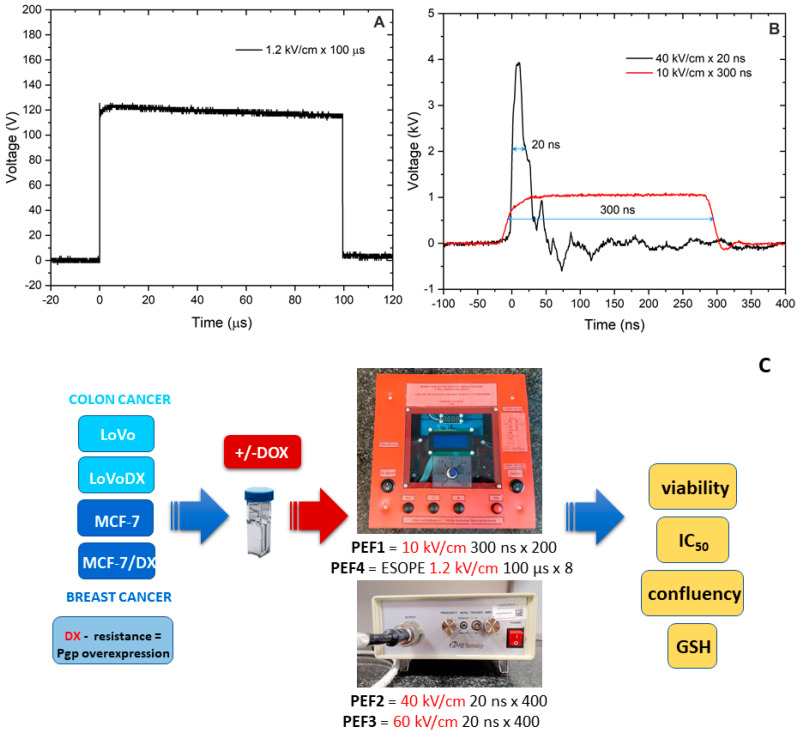
The waveform of (**A**) long- and (**B**) short-duration pulses and (**C**) graphical representation of the experimental setup.

For the experiments, the cells were trypsinized and centrifuged (5 min, 1000 rpm, MPW-341 Centrifuge with a stable rotor, MPW Med. Instruments, Warsaw, Poland). For each sample, 5 × 10^5^ of cells were resuspended in HEPES buffer (10 mM HEPES (C_8_H_18_N_2_O_4_S, cat. no.: H337, Sigma-Aldrich, Poznan, Poland), 250 mM sucrose (C_12_H_22_O_11_, Chempur, Piekary Slaskie, Poland), and 1 mM magnesium chloride (MgCl_2_, Sigma, M8266) in sterile MilliQ water. Electroporation protocols were combined with doxorubicin (DOX). In experiments, 2, 20, and 50 µM concentrations of DOX (Sigma-Aldrich, Poznan, Poland) were used according to prior studies [15,16]. DOX suspension was prepared in the same buffer as for electroporation alone. After pulsing, 10 min incubation at 37 °C was performed. Then, the cells were resuspended in the appropriate cell culture medium (DMEM or Ham’s F12) for further evaluation. Untreated controls were handled the same way as treated cells; all steps were performed simultaneously, and the same culture dishes were used (cuvettes, centrifugation tubes). For the MTT assay, cells were seeded into 96-well microculture plates (density: 4 × 10^4^ of cells in 200 µL of culture medium/well) (Nunc, Roskilde, Denmark). The procedure is presented in Figure 1.

### 2.6. Cellular Viability and IC50 Validation

The MTT assay was performed 24 or 72 h post-electroporation to determine the mitochondrial function of breast or colon cancer cells as a viability marker. First, the cells were incubated with 100 μL of the MTT (3-(4,5-Dimethylthiazol-2-yl)-2,5-Diphenyltetrazolium Bromide) reagent (Sigma-Aldrich, Poznan, Poland) at 37 °C for 1.5 h. Then, formazan crystals were dissolved with the addition of 100 μL of acidic isopropanol and mixed. The absorbance was measured at 570 nm using a multi-well plate reader (GloMax^®^ Discover, Promega, Madison, WI, USA). The results were presented as percentage values compared to the untreated controls. The obtained data served for IC50 calculation for DOX and DOX with PEF combination. Experiments have been repeated a minimum of three times in triplicate.

### 2.7. Cells’ Confluency and Volume

Cells were trypsinized and seeded on 96-well plate at density 10^4^ cells/well. After PEF protocols, simultaneously, the analysis of cells’ confluency was initiated every 1 h for 44 h. The measurements were performed on the live-cell imaging platform CELLCYTE X (Sygnis, Warsaw, Poland) placed in the cell culture incubator to assure 37 °C and 5% CO_2_. High contrast enhanced contour mode was used to help distinguish detected structures. The results were collected by Cellink Studio software (Sygnis, Warsaw, Poland). The measurements were performed in minimum 4 repetitions.

### 2.8. Quantification of Reduced Glutathione (GSH) Depletion

The GSH-Glo^TM^ assay (Promega, Madison, WI, USA) was used in the study. It is a sensitive luminescent assay for detecting and quantifying depletion of glutathione (GSH) in cells. This assay detects the conversion of a luciferin derivative into luciferin in the presence of GSH. The reaction is catalyzed by a glutathione S-transferase (GST) enzyme. Luminescent signal is proportional to the amount of glutathione present in cells. White 96-well plates were used in the study. Cells were seeded after PEF protocols at density 10^4^ cells/well, and after 44 h GSH was measured. The luminescence was measured using a multi-well plate reader (GloMax^®^ Discover, Promega, Madison, WI, USA). The values were normalized to the control untreated cells.

### 2.9. Fluorescent Imaging of Mitochondria and ROS

Two markers were used to visualize mitochondria and reactive oxygen species in colon and breast cancer cells after PEF and PEF-DOX. MitoTracker^TM^ Red CMXRos (λ_exc_. 579/λ_em_ 599, Thermo Fisher) for ROS detection and MitoTracker^TM^ Green FM (λ_exc_. 490/λ_em_ 516, Thermo Fisher) for mitochondria, DAPI was used for nuclei staining. Cells underwent PEF protocols as described in Section 2.4. Then, cells were seeded on cover microscopic slides. After 48 h, cells were fixed, and staining was performed according to the manufacturer’s protocols. FluoView FV1000 confocal laser scanning microscope (Olympus, Tokyo, Japan) was used for imaging.

### 2.10. Statistical Analysis

A one-way analysis of variance (ANOVA) was used to compare different treatments in all experiments. Tukey HSD and Sidak’s multiple comparison tests for evaluating the differences were used when ANOVA indicated a statistically significant result (*p* < 0.05 was considered statistically significant). The data were post-processed in GraphPad Prism 7.0 software (GraphPad Software, San Diego, CA, USA). All experiments were performed at least in triplicate, and the treatment results were expressed as mean ± standard deviation.

## 3. Results

**Figure 2:**
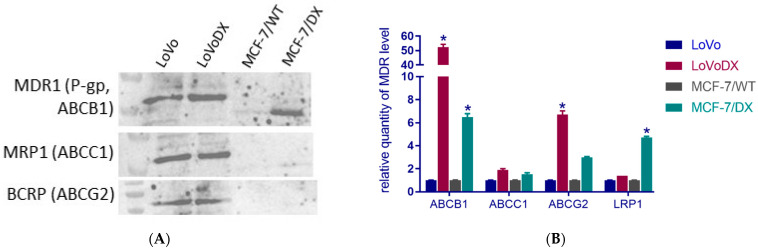
The level of MDR proteins in human and colon and breast cancer (**A**), relative quantity level of MDR genes expression (**B**). Asterisk (*) corresponds to statistically significant (*p* < 0.05) difference.

Firstly, we validated the level of resistance by western blot analysis and real-time PCR (Figure 2). Here, we used four cell lines where two revealed doxorubicin resistance. As we can observe, the most resistant cell was LoVoDX from colon cancer. Then, a high expression of ABCB1 and LRP1 was detected in MCF-7/DX cells.

### 3.1. Potentiation of Electrochemotherapy Using Nanosecond Bursts

**Figure 3:**
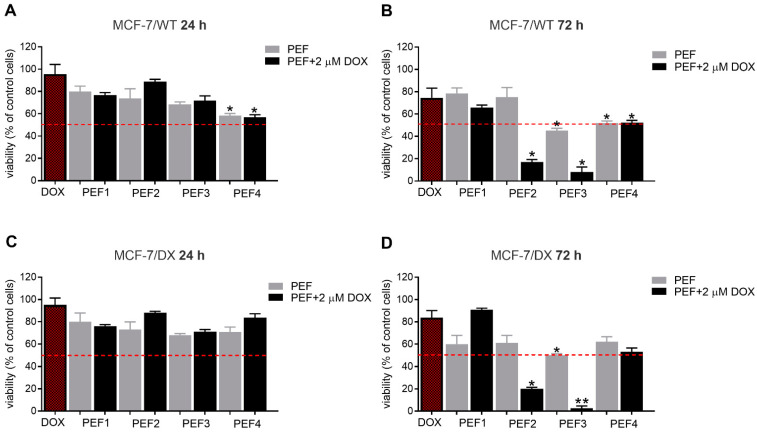
The viability of human breast adenocarcinoma cells exposed to electrical pulses without and with doxorubicin (DOX, C_DOX_ = 2 µM) after 24 h for (**A**) MCF-7/WT and (**C**) MCF-7/DX cells, and after 72 h for (**B**) MCF-7/WT and (**D**) MCF-7/DX cells. The following treatment protocols were used: PEF1—10 kV/cm × 300 ns × 200; PEF2—40 kV/cm × 20 ns × 400; PEF3—60 kV/cm × 20 ns × 400; PEF4—1.2 kV/cm × 100 µs × 8 (ESOPE); DOX—DOX treated only. All the data are normalized to untreated control. Asterisk (*) corresponds to (*p* < 0.05) or (**) to (*p* < 0.005) statistically significant difference.

**Figure 4:**
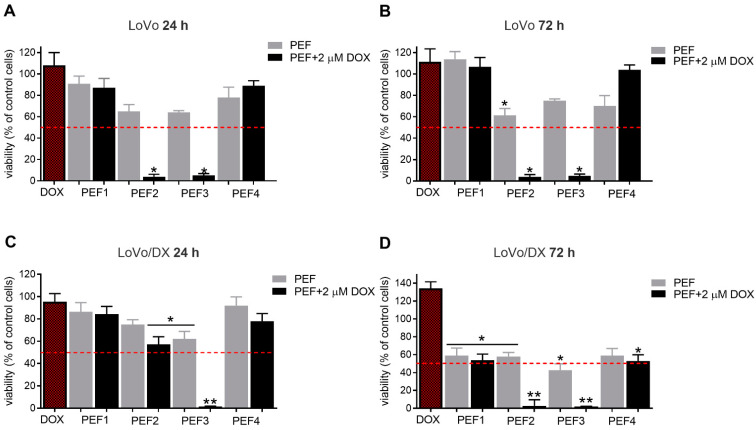
The viability of human colon adenocarcinoma cells exposed to electrical pulses without and with doxorubicin (DOX, C_DOX_ = 2 µM) after 24 h for (**A**) LoVo and (**C**) LoVoDX cells, and after 72 h for (**B**) LoVo and (**D**) LoVoDX cells. The following treatment protocols were used: PEF1—10 kV/cm × 300 ns × 200; PEF2—40 kV/cm × 20 ns × 400; PEF3—60 kV/cm × 20 ns × 400; PEF4—1.2 kV/cm × 100 µs × 8 (ESOPE); red bar DOX—DOX treated only. All the data are normalized to untreated control. Asterisk (*) corresponds to (*p* < 0.05) or (**) to (*p* < 0.005) statistically significant difference.

**Figure 5:**
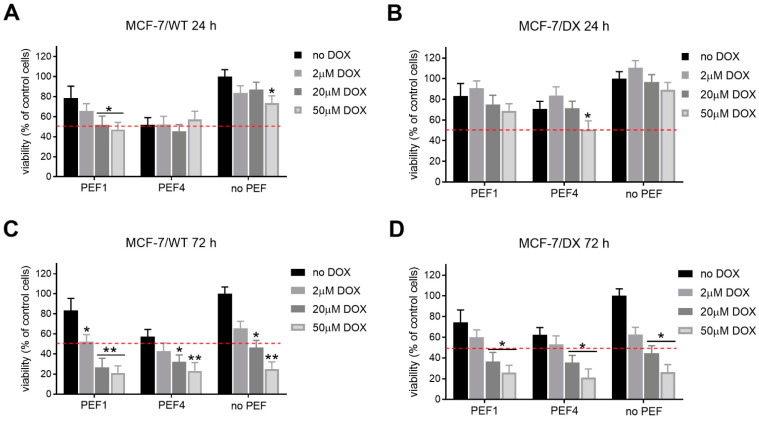
The viability of cells exposed to electrical pulses without (no DOX) and with doxorubicin (DOX, C_DOX_ = 2, 20 or 50 µM) after 24 h for MCF-7/WT (**A**) and MCF-7/DX (**B**) cells; 72 h for MCF-7/WT (**C**) and MCF-7/DX (**D**). The following treatment protocols were used: PEF1—10 kV/cm × 300 ns × 200; PEF4—1.2 kV/cm × 100 µs × 8 (ESOPE). All the data are normalized to untreated control. Asterisk (*) corresponds to (*p* < 0.05) or (**) to (*p* < 0.005) statistically significant difference.

**Figure 6:**
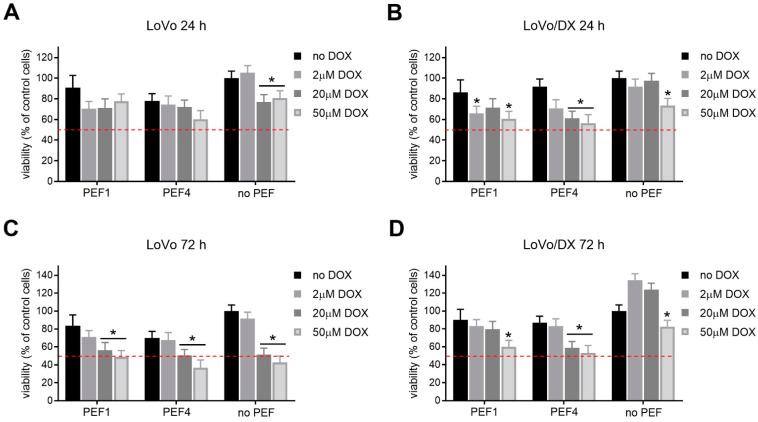
The viability of cells exposed to electrical pulses without (no DOX) and with doxorubicin (DOX, C_DOX_ = 2, 20 or 50 µM) after 24 h for LoVo (**A**) and LoVoDX (**B**) cells; 72 h for LoVo (**C**) and LoVoDX (**D**) cells. The following treatment protocols were used: PEF1—10 kV/cm × 300 ns × 200; PEF4—1.2 kV/cm × 100 µs × 8 (ESOPE). All the data are normalized to untreated control. Asterisk (*) corresponds to statistically significant (*p* < 0.05) difference.

Pulsed electric fields were used in combination with doxorubicin in four cell lines. Mitochondrial activity was evaluated 24 and 72 h post-treatment. The results for each cell line are summarized in Figure 3, Figure 4, Figure 5 and Figure 6. First, the effects of PEFs and 2 µM doxorubicin were investigated to determine the efficiency of various protocols. In all cases, the results were compared with the reference protocol (PEF4), which represents the standard European procedures in electrochemotherapy (ESOPE—1.2 kV/cm × 100 µs × 8).

As can be seen in Figure 3A,C, 24 h post-treatment, the human breast adenocarcinoma cell line responded differently to various PEFs. PEFs themselves inhibited the viability of the cells by 20–40%, with up to 20% differences between separate protocols (PEF1–PEF4). However, the differences were not statistically significant in most of the cases. PEF1 had the mildest cytotoxic effect (*p* < 0.05, MCF-7/WT cells), while PEF4 was most robust in both DOX-sensitive and DOX-resistant cell lines. In all the cases, the combination with 2 µM doxorubicin did not significantly reduce cell viability 24 h post-treatment. In DOX-resistant cells, a tendency of viability stimulation was observed (PEF2–PEF4). Figure 3B,D shows that 72 h post-treatment, PEF2 and PEF3 (20 ns pulse sequences) dramatically affected cell viability compared to other protocols when combined with DOX. This is particularly important since the PEF (without DOX) does not trigger a statistically significant viability decrease compared to the reference PEF4, which indicates a potentiation of electrochemotherapy as a synergistic treatment. The 60 kV/cm and 20 ns protocols (PEF3) were more effective after 72 h than the 40 kV/cm procedure, which is expected due to higher PEF intensity.

A similar analysis was performed for the colon adenocarcinoma cells exposed to electrical pulses without and with doxorubicin. The results are summarized in Figure 4A–D.

As can be seen in Figure 4, DOX-sensitive LoVo cells were almost completely inhibited using PEF2 and PEF3 protocols already after 24 h post-treatment. In turn, the viability of the LoVoDX cell line was slightly reduced after PEF2 (40 kV/cm) with DOX, and significantly reduced after PEF3 with DOX where 60 kV/cm was already above the threshold. Similar to breast adenocarcinoma, the PEF1 protocol was one of the weakest protocols independently on the cell line. Additionally, the PEF4 standard protocol did not affect cell viability, indicating different susceptibility of cancers to PEF. After 72 h, the ultrashort nanosecond protocols were significantly more cytotoxic (few percentages) than the conventional protocol or 300 ns protocol. The effect is synergistic and was not influenced by the PEF, only in the breast adenocarcinoma case.

### 3.2. The Effects of Doxorubicin Concentration in Electrochemotherapy

It was shown that nanosecond bursts (40 ns) trigger a significant synergistic response expected during electrochemotherapy. However, we wanted to test if the increase in DOX concentration can compensate for the weak effectiveness during PEF1 and PEF4. The concentration was increased from 2 μM to 20 and 50 μM, respectively. The results for breast adenocarcinoma cell lines are summarized in Figure 5.

As can be seen in Figure 5, 24 h post-treatment, the effectiveness of electrochemotherapy can hardly be improved by the dramatic increase in DOX concentration. The differences are not statistically significant in most cases, with a maximum average improvement of up to 20%. As expected, the DOX-resistant cell line was more resistant to treatment (2–20 μM concentration range). The observations after 24 h are not sufficient to characterize the cytotoxic activity of the treatment. Thus, the cellular response after 72 h was also evaluated (Figure 5C,D). It was shown that an increase in DOX concentration could be used to manipulate the treatment efficacy.

A similar analysis has been performed for colon adenocarcinoma cells. The results are summarized in Figure 6.

It can be seen that after 24 h, the increase in DOX concentration does not improve the efficacy of electrochemotherapy and mainly affects only the treatment outcome without PEF. The differences between the DOX-sensitive and DOX-resistant cell lines are negligible. However, the cytotoxic efficacy improves after 72 h, mainly in LoVo cells. Thus, we can conclude that DOX-PEF is more beneficial in the case of breast cancer cells than in colon cancer cells, with the weakest effect in resistant colon cancer cells. The obtained results enabled the evaluation of IC50 for DOX combined with PEF protocols, which is shown in Table 1.

We can see that PEF decreased IC50 in all cases. The most significant decrease was obtained for MCF-7/WT cells, then for MCF-7/DX, and similar values were obtained for both colon cancer cell lines.

**Figure 7:**
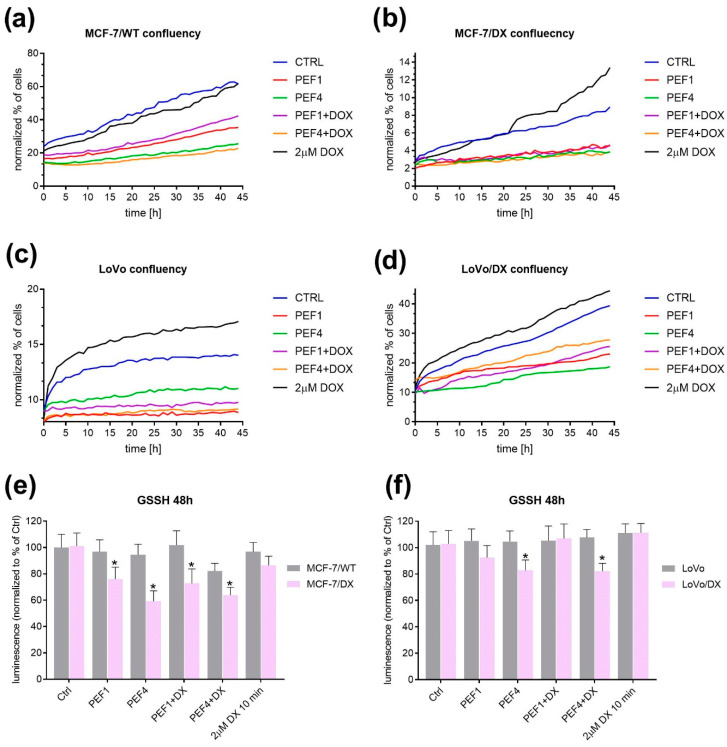
The analysis of the effects after exposure to electrical pulses without and with doxorubicin (DOX, C_DOX_ = 2 µM) on cells’ confluency analyzed during 44 h for breast cancer MCF-7/WT (**a**) and MCF-7/DX (**b**) cells, and colon cancer LoVo (**c**) and LoVoDX (**d**) cells; the evaluation of reduced glutathione (GSSH) in breast cancer (**e**) and colon cancer (**f**) cells. The following treatment protocols were used: PEF1 —10 kV/cm × 300 ns × 200; PEF4—1.2 kV/cm × 100 µs × 8 (ESOPE). All the data are normalized to untreated control. Asterisk (*) corresponds to statistically significant (*p* < 0.05) difference.

Furthermore, the effect of PEFs with selected DOX concentration (2 µM) on cell confluency was evaluated within 44 h (Figure 7). In the case of MCF-7/WT cells, the weakest increase in cells’ confluency was observed after the exposure to PEF4 and PEF4-DOX and then PEF1 and PEF1-DOX. Control cells and DOX treated maintained a similar level; cells’ confluency increased in time. However, in the case of DOX alone, the increase was more evident, which might point to strong drug resistance. In the case of MCF-7/DX, both protocols affected cells’ confluency equally. Moreover, in the case of colon cancer, both PEF protocols similarly affected cells. DOX combined with PEF also increased cells’ confluency but significantly slower.

The reduced glutathione (GSSH) level was measured in MCF’s and LoVo’s cell lines and presented in Figure 7e,f. The decrease in GSSH was observed only in resistant cells, in MCF-7/DX after both PEF protocols, and in LoVoDX after PEF4, which had the strongest effect.

**Figure 8:**
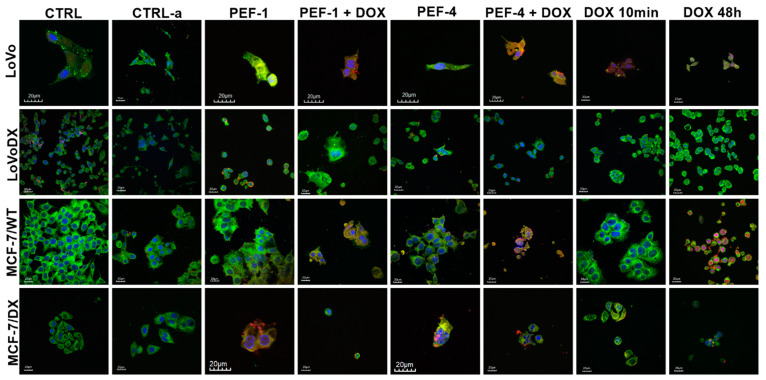
The visualization of mitochondria (green) and ROS (red) in colon and breast cancer cells after PEF1 and PEF4 with/without doxorubicin (DOX, C_DOX_ = 2 µM).

In the next stage, the effect of PEFs on mitochondria and reactive oxygen species release was visualized. Figure 8 shows the presence of reactive oxygen species in LoVo cells after PEF1 and PEF4 with DOX. It can also be seen that the treatment affected cell morphology, i.e., cell shrinkage and reduced cells number. In the case of LoVo/DX cells, both PEF protocols provoked ROS release. MCF-7/WT released ROS after both PEFs with DOX. Cells incubated 48 h with DOX show strong, nuclear accumulation of doxorubicin, but cells’ morphology is not strongly affected compared to PEF treatment. In the case of MCF-7/DX cells, ROS were detected after PEF1, PEF4, and PEF4 + DOX. Moreover, the combined treatment strongly reduced cell number, which is consistent with the confluency results.

## 4. Discussion

Drug resistance is still not a solved problem in the anticancer protocols. Researchers are still attempting to overcome this phenomenon using various chemical or physical methods [24,25]. Here, we have used drug-resistant models of colon and breast cancer. In breast cancer, we deal with drug resistance acquired through chemotherapy [26], while in colorectal cancer, the problem is more severe because this type of tumor is often characterized by primary resistance [24]. Thus, overcoming the cell membrane barrier using physical methods such as electroporation [18] seems to be a promising solution. Our study implements various parameters of the pulsed electric field (PEF) to enhance doxorubicin delivery and effects. As was previously proved, doxorubicin can be effectively supported by microsecond pulses in colon cancer [15] or breast cancer [16]. In this study, the first stage examined the potentiation of electrochemotherapy using nanosecond bursts in combination with doxorubicin in four cell lines. Our result revealed that 72 h effects of PEF2 and PEF3 with DOX (20 ns pulse sequences) significantly inhibited cell viability. We could observe a potentiation of electrochemotherapy as a synergistic treatment. Moreover, 60 kV/cm and 20 ns protocols were more effective than the 40 kV/cm procedure, which is expected due to higher PEF intensity. Our previous study also confirmed that the ESOPE protocol (here PEF4) combined with doxorubicin is effective in human breast cancer [16] and demonstrated that the application of electric pulses affects cell morphology [16] and increases cell membrane permeability [16,27,28,29]. There was also observed an increase in reactive oxygen species, abnormalities in mitochondria, and an increase in intracellular calcium [16]. It should be noticed that electrochemotherapy requires optimization of drug concentration. The efficacies are comparable with no PEF treatments, which is an extremely important result. The success of DOX-based electrochemotherapy is pulse dependent and is occurrent in the ultrashort pulse range, while conventional protocols are no longer competitive. The same assumption and tendency were observed for both the DOX-sensitive and DOX-resistant cell lines.

In this study, the effect of PEFs on oxidative stress markers (reduced glutathione and ROS) was also determined. As was demonstrated, PEF protocols decreased GSSH and stimulated ROS release in resistant cells. Our previous study showed that electroporation combined with caffeic acid phenethyl ester could also decrease the glutathione ratio in human melanoma cells [30]. In cancer therapy, doxorubicin delivery is problematic, particularly in drug-resistant cancers, which overexpress MDR-related proteins in cell membranes. These proteins work in the same way as pumps and block or effectively eliminate molecules of cytostatic drugs [31]. Our previous study proved by a rhodamine-123 efflux assay that MDR1 activity decreased after nanosecond electroporation in MCF and LoVo cells [32]. The application of PEFs contributes to the reversible or irreversible lipid membrane reorganization [18] and changes in MDR pumps’ activity. This effect also induces oxidative stress, which depends on the PEF’s parameters [33,34]. These factors contribute to a better cytostatic activity, which can enter the cell and lipid membrane, and MDR proteins are not a barrier. Then, doxorubicin can contribute to the intensified oxidative stress by various mechanisms, e.g., inactivation of ROS-related antioxidative enzymes or disruption of mitochondrial electron transport chains [35]. Thus, the combination of PEFs and DOX-induced oxidative stress can lead to a more efficient elimination of cancer cells.

Currently, cisplatin and bleomycin are two approved drugs that can be effectively used in electrochemotherapeutic procedures [36]. The obtained results highlight the potential of pulsed electric fields in doxorubicin delivery using only 10 min exposure to the low drug concentration. The limited time of exposure and low drug concentration might also be beneficial in terms of cardiotoxicity and hepatotoxicity. For example, Srimathveeravalli et al. demonstrated that reversible electroporation can assist liposomal doxorubicin (DOX) delivery to tumors through transfection and can induce changes in vascular permeability [6]. Interestingly, Meschini et al. suggested DOX administration to LoVoDX cells after electric pulses and increased intranuclear DOX accumulation [37]. Our previous study on colon and gastric cancers also confirmed anticancer effects of EP with DOX, which disturbed the ultrastructure significantly and triggered changes in P-glycoprotein expression [15]. Drug-resistant-related proteins and genes strongly hinder drug transport; thus, physical methods such as electroporation, in particular in the nanosecond range, seem to be a good support in chemotherapy.

## 5. Conclusions

Our results indicate the high potential of 20 nanosecond pulses and high voltage combined with doxorubicin against breast and colon cancer, in particular with drug resistance. The efficacy of protocols using longer pulse duration, such as 300 ns and 100 µs, can be enhanced by increasing the doxorubicin concentration. However, even 2 µM DOX combined with PEF provoked ROS release, decreased the reduced glutathione (GSSH) level, and affected confluency and cell viability. PEF application seems promising because of the short time protocols compared to the classical anticancer treatment and enabled a significant decrease in active drug concentration. The proposed study of sensitive and resistant cells can be efficiently transposed to the other models used with electroporation research. However, when combined with chemotherapy, the variety of PEF’s parameters requires optimization for the particular cancer type.

## Figures and Tables

**Table 1:** IC50 values that reduce cell proliferation by 50% calculated and represented as micromolar concentration for DOX and PEF + DOX exposure after 24 and 72 h. PEF1 and PEF4 (ESOPE) protocols were selected.

24 h	MCF-7/WT	MCF-7/DX	LoVo	LoVo/DX
DOX	62.06	139.09	93.69	97.67
PEF1-DOX	18.55	88.85	91.89	97.85
PEF4-DOX	7.48 *	51.25	85.99	43.51
**72 h**				
DOX	18.69	16.31	96.51	94.26
PEF1-DOX	0.67 *	10.41	43.67	48.14
PEF4-DOX	0.64 *	3.19 *	26.85	21.79

* *p* < 0.05.

## Data Availability

Data sharing not applicable.
